# Bipolar Disorder is associated with the rs6971 polymorphism in the gene encoding 18 kDa Translocator Protein (TSPO)^☆^

**DOI:** 10.1016/j.psyneuen.2013.07.007

**Published:** 2013-11

**Authors:** Alessandro Colasanti, David R. Owen, Detelina Grozeva, Eugenii A. Rabiner, Paul M. Matthews, Nick Craddock, Allan H. Young

**Affiliations:** a Division of Brain Sciences, Imperial College, London, United Kingdom; b Imanova Centre for Imaging Sciences, Hammersmith Hospital Campus, London, United Kingdom; c Centre for Neuroimaging Sciences, Institute of Psychiatry, King's College London, United Kingdom; d Neurosciences, GlaxoSmithKline, London, United Kingdom; e MRC Centre for Neuropsychiatric Genetics and Genomics, Cardiff University, Cardiff, United Kingdom; f Centre for Affective Disorders, Department of Psychological Medicine, Institute of Psychiatry, King's College, London, United Kingdom

**Keywords:** Translocator Protein, Cortisol, HPA, Neurosteroids, rs6971, Genome-wide, WTCCC, Polymorphism, Mitochondria, Bipolar Disorder

## Abstract

TSPO mediated transport of cholesterol into the mitochondrion is a necessary step in steroid synthesis. The rs6971 polymorphism in the *TSPO* gene causes an amino acid substitution (Ala147Thr) within the transmembrane domain where the cholesterol-binding pocket is located, and has been shown to affect the steroidogenic pathway. We report a nominal association between this *TSPO* polymorphism and the diagnosis of Bipolar Disorder in both the genome-wide dataset of the Wellcome Trust Case–Control Consortium and the Psychiatric Genome-Wide Association Study Consortium Bipolar Disorder group (OR = 1.11, *p* = 0.007; OR = 1.10, *p* = 0.011, respectively). We propose that the amino acid substitution affects hypothalamic–pituitary–adrenal (HPA) regulation, and hence may predispose to Bipolar Disorder. This supports the hypothesis that HPA dysregulation has a causal role in Bipolar Disorder, and is not just a consequence of the disease.

## 1 Introduction

The aetiopathogenesis of Bipolar Disorder (BD) involves both environmental and genetic factors. The hypothalamic–pituitary–adrenal (HPA) axis is a physiological system which is subject to both these influences and is dysregulated in BD both during acute illness and as an enduring correlate (Watson et al., 2004). Furthermore, increased daytime cortisol secretion, which reflects HPA axis dysfunction, has been also observed in the adolescent offspring of parents with BD and persists into young adulthood (Ellenbogen et al., 2006). However, it is currently unclear whether the HPA axis dysfunction is a cause or consequence of BD.

The rate-limiting step in the synthesis of all steroids, including those involved in the HPA axis, is the delivery of cholesterol from the outer (OMM) to the inner mitochondrial membrane (IMM). At the IMM, cholesterol is converted to pregnenolone, the common precursor for all steroids, by the cytochrome P450 enzyme CYP11A1, which cleaves the cholesterol side chain. The delivery of cholesterol to the IMM is dependent upon the function of the 18 kDa Translocator Protein (TSPO), located on the OMM, which binds cholesterol with high affinity and imports it across the membrane. If TSPO is knocked down or pharmacologically inhibited, steroid synthesis ceases (Hauet et al., 2005). A common single nucleotide polymorphism (rs6971) in the *TSPO* gene leads to an amino-acid substitution, Ala147Thr, which dramatically alters the affinity with which TSPO binds drug ligands (Owen et al., 2012). As cholesterol also binds TSPO in the same transmembrane domain, we hypothesised that this substitution may impair the ability of TSPO to bind or import cholesterol, and hence may affect steroid synthesis and HPA function.

The hypothesis that HPA dysfunction plays a causal role in BD pathophysiology would be supported by a finding that a genetic variant, which leads to a dysregulated HPA function, is associated with diagnosis of BD. To test this we investigated the association between rs6971 and a diagnosis of BD in the datasets of the Wellcome Trust Case–Control Consortium (WTCCC) and the Psychiatric Genome-Wide Association Study Consortium Bipolar Disorder group (PGC-BD).

## 2 Materials and methods

This is a large-scale case–control study which aims to test the association between the SNP: rs6971 and BD. We analysed the cases reported in the BD subset of the WTCCC genome-wide association study (GWAS), which comprises 1868 BD cases and 2938 controls (WTCCC, 2007). The study procedures were conducted in accordance with the Declaration of Helsinki and were carried out after all subjects provided informed written consent.

As rs6971 was not directly genotyped in the WTCCC study, the data were imputed from nearby SNPs using the software IMPUTE2 according to the method of Howie et al. (2009). The SNP rs138911, in linkage disequilibrium with the SNP of interest, rs6971, was genotyped to further infer the allele of the SNP rs6971.

The analysis of the association between rs6971 and diagnosis of BD was also examined using the meta-analytic data of the PCG-BD, which combines primary genotype data from large GWAS datasets. The PGC-BD dataset is made up of 7481 cases and 9250 controls, and also comprises the WTCCC data (Sklar et al., 2011). For the PCG-BD, rs6971 was imputed using the software BEAGLE (Browning and Browning, 2007).

## 3 Results

In this imputation-based analysis of the WTCCC sample we observed an underrepresentation of the minor allele (Thr147) of the SNP rs6971 in BD, with a frequency of 31.1% in BD and 33.4% in controls (OR 1.1148; *p* = 0.007). The frequencies of genotypes of the SNP rs138911, in linkage disequilibrium with rs6971 (LD *R*^2^ = 0.77), in BD patients were T/T: 6.3%; T/G: 40%; G/G: 53.6%; whilst the frequencies of genotypes in controls were T/T: 8%; T/G: 41.3%; G/G: 50.7% (Table 1).

The replication analysis from the PGC-BD data showed a similar association between the SNP rs6971 and BD diagnosis (Table 1).

## 4 Discussion

Our data indicate a nominal association between the rs6971 SNP in the *TSPO* gene and the diagnosis of BD. Evidence for this is provided by the imputed association in the WTCCC data between rs6971 and BD diagnosis, as well as by the association between BD and the directly genotyped rs138911 SNP, in linkage disequilibrium with rs6971. The observation of the association between rs6971 and BD diagnosis was replicated in the larger, partially overlapping, PGC-BD cohort.

These findings did not reach genome-wise significance in either sample, and although they provide suggestive evidence for an association of rs6971 with BD, a replication of these observations is required. It is important to note, however, that we tested a single specific hypothesis and pre-specified both phenotype and SNP a priori, making comparisons with statistical approaches for GWAS studies difficult. We hypothesised that rs6971 would be associated with HPA dysregulation. Hence, we tested for an association with BD, the psychopathological condition where HPA dysfunction is most evident and is independent of transient disease state (Watson et al., 2004). Whilst depression, schizophrenia, and Post Traumatic Stress Disorder (PTSD) have also been associated with HPA alterations, dysfunction in depression is dependent on disease state, in schizophrenia is much less marked relative to BD (Sharma et al., 1988), and in PTSD may represent a marker of exposure to trauma rather than a specific mechanism of vulnerability for PTSD (Morris et al., 2012). Hence association with these phenotypes was not tested.

This hypothesis was based on our in vitro data. Binding of TSPO to synthetic and endogenous ligands is modulated by polymer formation (Delavoie et al., 2003), interaction with a multimeric complex, redistribution of TSPO molecules (Boujrad et al., 1996), and other factors. However, we have shown that in human samples the predominant determinant of TSPO binding affinity is the rs6971 polymorphism, although this has only been tested in synthetic ligands hitherto (Owen et al., 2012). The amino acid residue which is altered by rs6971 (residue 147) sits within the 5th transmembrane domain of the TSPO, which is the same location as the cholesterol binding pocket (Li and Papadopoulos, 1998). As the binding affinity of all synthetic ligands known to bind in the 5th transmembrane domain is affected by rs6971, we hypothesised that this substitution may alter cholesterol handling by TSPO. The results we present here are consistent with this hypothesis.

Although we chose to test for an association with BD on the basis that rs6971 might impact on HPA dysregulation, it is also possible that the link between rs6971 SNP and BD is independent of the HPA axis and instead mediated by differences in neurosteroids. Whilst synthesis and control of neurosteroids differs from peripheral steroids, the rate-limiting step in both is TSPO-mediated movement of cholesterol across the OMM. Hence, an altered cholesterol transport regulation may potentially affect the biosynthesis of neurosteroids, which have been directly implicated in the pathophysiology of BD (Carta et al., 2012). Interestingly, an association between rs6971 and alteration in peripheral pregnenolone production has been reported (Costa et al., 2009a). In healthy volunteers, the heterozygous and Thr147 homozygous genotype show significantly lower pregnenolone levels compared with the Ala147 homozygous, indicating a dominant effect of the Thr147 allelic variant. This suggests that the presence of at least one copy of the Thr147 allele may impair cholesterol translocation efficacy and peripheral steroid production. However, the downstream effect this has on cortisol production, HPA function, or neuroactive steroids’ function, is unknown.

It should be noted that a study in a Japanese sample (n. cases = 94; n. controls = 359) found no association of rs6971 with BD (Kurumaji et al., 2001). However, whilst the minor allele frequency is 30% in Europeans, it is only 4% in the Japanese population. Hence, this study included no Thr/Thr subjects and only 17 heterozygotes and so had limited power to detect any association. Another study in a Caucasian sample (Costa et al., 2009b) found that rs6971 SNP was associated to the presence of separation anxiety but not to a diagnosis of depression (n. unipolar or bipolar depressed patients: 190; n. controls = 182). The discrepancy between these and our findings may depend on the different size of the sample and on the different disease target, possibly suggesting that the association is specific for BD. Another possibility is that an association of rs6971 with the intermediate phenotype ‘separation anxiety’ may underline the observed association with BD in our sample. We cannot test this in our study however evidence of high prevalence of separation anxiety in BD seems to support this hypothesis (Sala et al., 2010).

There is growing interest in the role of TSPO in the pathophysiology of anxiety/affective disorders, based on alterations in neurosteroid concentrations in these diseases and experimental data showing TSPO targeting drugs might be efficacious in anxiety disorders (Rupprecht et al., 2010). Our findings suggest that TSPO may have a causal role in BD and hence may also be a drug target.

A caveat of our study is that the rs6971 SNP was not directly genotyped and the association was inferred by imputation. Although the imputation methods we used to infer from missing data are well established and supporting evidence indicates they are accurate (Browning and Browning, 2007; Howie et al., 2009), the fact that our findings derived from statistical inference is a disadvantage. Furthermore, the observed association only reached nominal significance for genome-wise studies. By using uncorrected *p*-values, up to 1% of SNPs in similarly sized GWAS studies would be expected to show the level of association observed for rs6971. However, as we tested a unique specific a priori hypothesis, we feel that the observed association is a meaningful and important finding. Finally the two datasets, WTCCC and PGC-BD are partially overlapping, as the WTCCC dataset comprises 25% of the PGC-BD cases. Although it is possible the association observed in the PGC-BD dataset is highly influenced by the WTCCC cases, we observed that the strength of the association of rs6971 SNP to BD diagnosis is similar between the two samples (OR = 1.11 and 1.10, respectively), despite only 25% overlap between cases. This suggests that the association in the PCG-BD cases exists independently of the WTCCC cases. Based on the above-described limitations, a replication of the study of this association in an independent sample is warranted to corroborate our findings.

In conclusion, our findings suggest an association between BD and the rs6971 polymorphism in the TSPO *gene*, which may alter steroid synthesis or its regulation. If this will be confirmed by replication, it would provide the first direct evidence that HPA dysregulation may have a causal role in BD and is not simply a consequence of the disease.

## Conflicts of interest

There are no conflicts of interest that might bias this work.

## Figures and Tables

**Table 1:** Allele and genotype distributions in WTCCC BD and PGC-BD cases and controls.

	SNP ID	*N*	Allele frequency		*p*-Value	OR	Genotype [genotype counts, frequency]		
			A1	A2			A1	A1/A2	A2
WTCCC	rs6971	*Cases* (1868)	31.1%	68.9%	0.007	1.1148	AA [173; 9.3%]	AG [815; 43.6%]	GG [880; 47.1%]
	*Imputed data*	*Controls* (2938)	33.4%	66.6%			AA [332; 11.3%]	AG [1300; 44.2%]	GG [1306; 44.4%]
	rs138911^a^	*Cases* (1863)	26.3%	73.7%	0.015	1.1203	TT [118; 6.3%]	TG [746; 40%]	GG [999; 53.6%]
	*Genotyped data*	*Controls* (2930)	28.6%	71.4%			TT [233; 8%]	TG [1211; 41.3%]	GG 1486; 50.7%]

PGC-BD	rs6971	*Cases* (7481)	29.6%	70.4%	0.011	1.1028			
	*Imputed data*	*Controls* (9250)	31.7%	68.3%					

*Abbreviations*: A1: allele 1; A2: allele 2; OR: odds ratio; LD: linkage disequilibrium.

a LD with rs6971 (*R*^2^ = 0.77).
